# Detecting COVID-19 from Breath: A Game Changer for a Big Challenge

**DOI:** 10.1021/acssensors.1c00312

**Published:** 2021-04-07

**Authors:** Giorgia Giovannini, Hossam Haick, Denis Garoli

**Affiliations:** † Empa, Swiss Federal Laboratories for Materials Science and Technology, Laboratory for Biomimetic Membranes and Textiles, Lerchenfeldstrasse 5, CH-9014, St. Gallen, Switzerland; ‡ Department of Chemical Engineering, Biomedical Engineering & The Russell Berrie Nanotechnology Institute (RBNI) Technion − Israel Institute of Technology, Haifa 32000003, Israel; § Istituto Italiano di Tecnologia, via Morego 30, I-16163, Genova, Italy; ∥ Faculty of Science and Technology, Free University of Bozen, Piazza Università 5, 39100 Bolzano, Italy

**Keywords:** COVID-19, diagnostics, sensor, breath, virus, volatile organic compounds, VOCs, detection

## Abstract

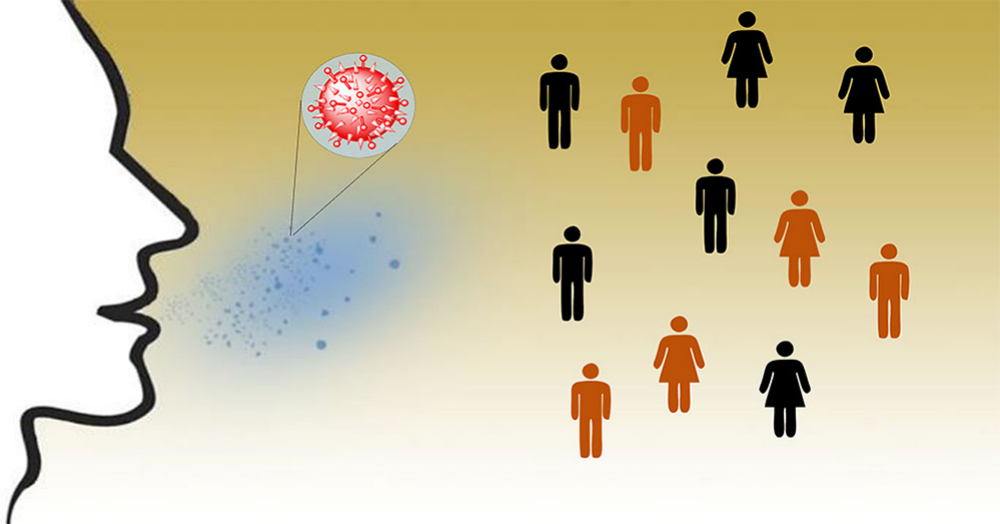

Coronavirus disease 2019 (COVID-19) is probably the most commonly heard word of the last 12 months. The outbreak of this virus (SARS-CoV-2) is strongly compromising worldwide healthcare systems, social behavior, and everyone’s lives. The early diagnosis of COVID-19 and isolation of positive cases has proven to be fundamental in containing the spread of the infection. Even though the polymerase chain reaction (PCR) based methods remain the gold standard for SARS-CoV-2 detection, the urgent demand for rapid and wide-scale diagnosis precipitated the development of alternative diagnostic approaches. The millions of tests performed every day worldwide are still insufficient to achieve the desired goal, that of screening the population during daily life. Probably the most appealing approach to consistently monitor COVID-19 spread is the direct detection of SARS-CoV-2 from exhaled breath. For instance, the challenging incorporation of reliable, highly sensitive, and cost-efficient detection methods in masks could represent a breakthrough in the development of portable and noninvasive point-of-care diagnosis for COVID-19. In this perspective paper, we discuss the critical technical aspects related to the application of breath analysis in the diagnosis of viral infection. We believe that, if achieved, it could represent a game-changer in containing the pandemic spread.

The last year has been critical for the whole world. The unexpected COVID-19 pandemic completely changed daily life of most of the population. Every day we talk about the number of confirmed cases, deaths, and hospitalizations, and discussions are constantly being held on how to improve the testing efficiency for COVID-19, to better understand and contain the disease spread.

The standard methods to test for COVID-19 rely on polymerase chain reaction (PCR) technologies. PCR is well-known to ensure high accuracy and high specificity (e.g., low levels of false positives and negatives). Yet, the efficiency of this approach is hindered by the slow delivery of the results, mostly 1 or 2 days after sampling. Rapid tests, typically based on lateral flow assays or ELISA technologies, therefore are routinely used as prescreening methods. The results of these tests are available in 10–30 min, and their sensitivity is up to 90%.^1^ Both detection techniques—rapid antigenic tests and sensitive molecular tests—have limitations in terms of testing procedures. The first is that they require trained personnel and properly equipped test sites, something that involves challenges with the operational logistics and product supply chains for the enormous number of tests per day in every country. The second is that the analysis is of nasopharyngeal and oropharyngeal specimens. This procedure is unpleasant for the patient, misses a standard sampling, and could miss areas with high viral loads during the swabbing, something that could lead to false-negative test results.

As discussed below, several other methods and devices have been proposed or are now under investigation. However, the majority of them are being assessed using materials extracted from blood, nasal or oral swabs, sputum, and, more recently, feces (interestingly, urine cannot be used because it is rare to find SARS-CoV-2 virus in it).^2−4^ It is now well-known that the two major ways of COVID-19 spread are airborne and contact infections/diffusion.^5,6^ This seems to be due to the high resistance of the virus once in aerosol droplets expelled from infected persons (Figure 1).

**Figure 1:**
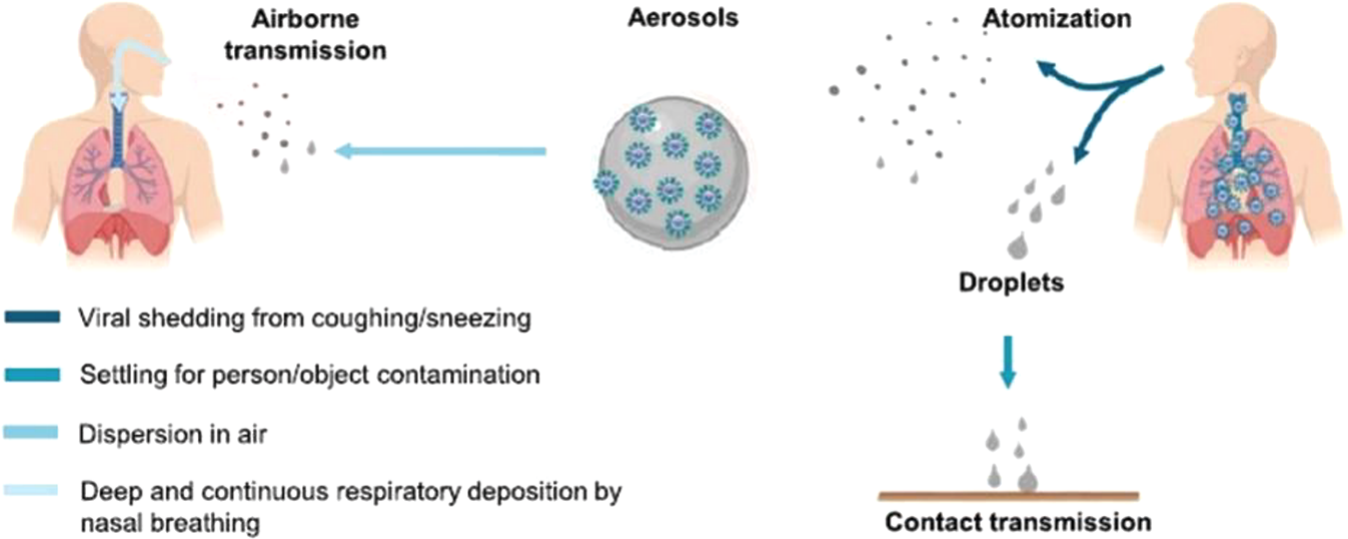
Transmission of COVID-19. Human atomization of viruses arises from coughing or sneezing of an infected person, producing virus-containing droplets (>5 μm) and aerosols (<5 μm). Virus transmission from person to person occurs through direct/indirect contact and airborne aerosol/droplet routes. Large droplets mainly settle out of the air to cause person/object contamination, whereas aerosols efficiently disperse in air. Direct and airborne transmissions occur in short-range and extended distance/time, respectively. Inhaled airborne viruses are deposited directly on to the human respiration tract. Figure adapted with permission from ref (6), 2020, editor of the National Academy of Sciences.

Several investigations and strategies to mitigate infection have been proposed, in particular, ones related to social distance and the fundamental use of face masks.^7,8^ Among the others, Cowling et al.^8^ reported on the detection of SARS-CoV-2 virus directly from exhaled breath and coughs in patients with acute respiratory illness. The study was intended to demonstrate the efficacy of face masks in preventing virus diffusion, but at the same time, it suggested the plausibility of direct detection of COVID-19 from breath. This approach is now attracting significant interest to other viral diagnostics.^9^ Several reviews have been published on breath analysis.^10−13^ In this Perspective, we will focus on breath analysis for COVID-19 diagnostics. As discussed below, to date it has been possible to demonstrate the direct detection of COVID-19 virus from exhaled breath only by using specific devices that can collect and condense exhaled breath for several minutes, and by using this condensate to extract the virus and follow the standard PCR based routine. Amplification-free detection has yet to be demonstrated. Moreover, it has been extensively demonstrated that the virus induces the cells to produce metabolites, which leads to volatile organic compounds (VOCs) being exhaled. These VOCs can be targets of breath diagnostics and used to assess health status without being invasive for patients. Recent reports have been on viral-associated breath VOCs for both rhinovirus^14^ and seasonal influenza respiratory tract infections.^15^ More recently, they have also tentatively linked specific breath VOCs with SARS-CoV-2 infections.^9,16,17^ These pioneer studies suggest a clear correlation between specific VOCs and COVID-19 infection. The procedures used to collect exhaled breath and the low reproducibility of the results show that a lot of work is still needed to make exhaled breath analysis a robust method of detection. In this perspective paper, we discuss how exhaled breath analyses could be a potential game-changer for the prescreening of virus infection, in particular, for the current COVID-19 pandemic. We will discuss the issues related to COVID-19 detection and sensing, and try to correlate the recent findings on COVID-19 diffusion mechanisms considering the great challenge of directly detecting SARS-CoV-2 from the air and exhaled aerosols and breath.

## State-of-the-Art in Sensing COVID

The abundance of publications associated with the SARS-CoV-2 outbreak is indicative of the intense effort by research institutes and pharmaceutical industries to gain knowledge about this newly identified virus, as well as to develop vaccines, therapeutics, and diagnostics. So far, massive-scale testing has been the main strategy adopted for the containment of the COVID-19 pandemic, but the analytical laboratories have been overloaded with requests and the test supply was insufficient.^18−20^ To maximize test availability, the US FDA has approved diagnostic tools with a simplified procedure granted by the Emergency Use Authorization (EUA). Many authors have extensively reviewed the commercialized devices highlighting their sensitivity and time required for the results.^21,22^ A massive number of methods are available in the literature proposing novel approaches to develop rapid, highly sensitive, cost-efficient, and easy-to-use point-of-care devices for COVID-19 diagnosis.

**Figure 2:**
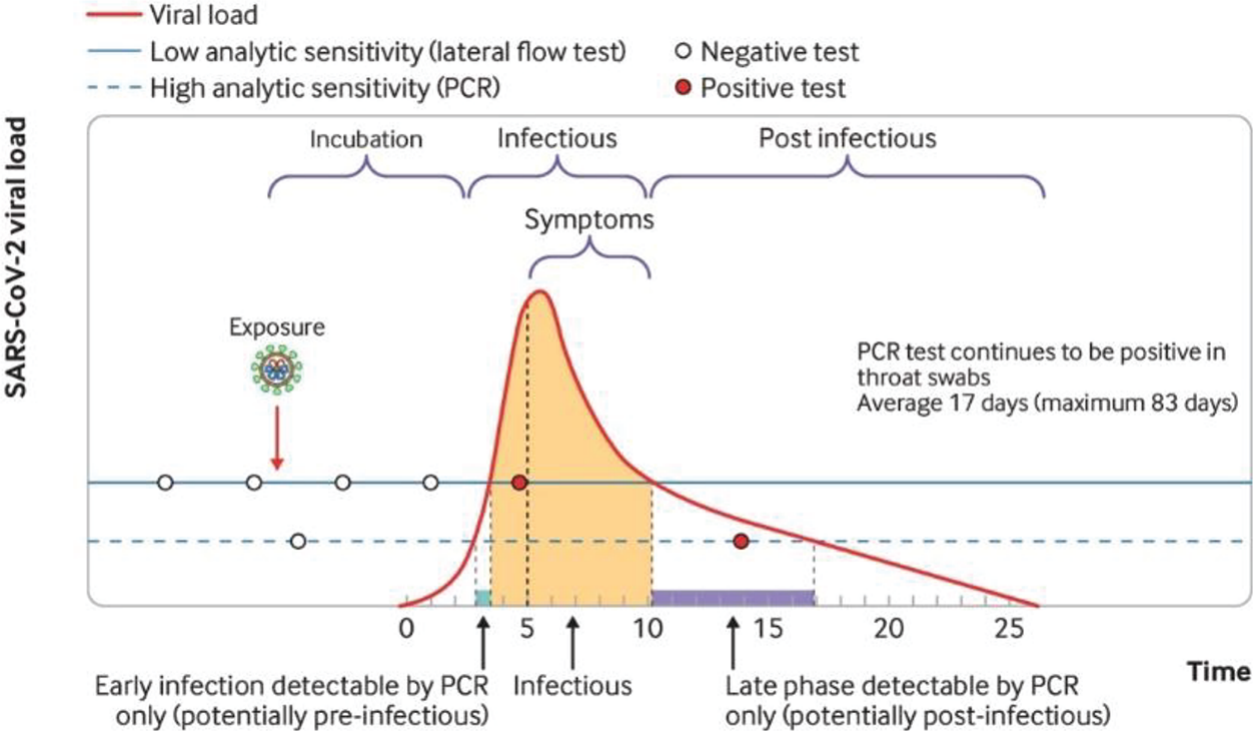
High-frequency testing with low analytic sensitivity versus low-frequency testing with high analytic sensitivity. A person’s infection trajectory (blue line) is shown in the context of two surveillance regimens (circles) with different analytic sensitivity. Higher frequency testing is more likely to test in the infectious window. Therefore, although both testing regimens detect the infection (orange circles), the high-frequency lateral flow test is more likely to detect it during the transmission window (shading), despite its lower analytic sensitivity. The figure is not an accurate representation of exactly when a positive test is likely to signify that a case is infectious. Adapted with permission from ref (1). BMJ Publishing Group.

Molecular tests used for confirming COVID-19 are considered to be the gold standard for SARS-CoV-2 testing, whereas serological tests are used for antibody detection. The three main detection methods are (i) identification of the viral gene region through nucleic acid amplification techniques (PCR), gene sequencing,^23^ and CRISPR-based nucleic acid detection;^24^ (ii) recognition of antibodies (IgM and IgG) produced to the viral infection (serological tests); and (iii) detection of specific SARS-CoV-2 antigens (i.e., spike, envelope, and nucleotide proteins). Each of these methods has pros and cons that have been critically reviewed.^25,26^ For instance, identification approaches of RNA/DNA require sophisticated devices and trained personnel. These protocols increase the occurrence of human errors during sample handling and analysis. Moreover, the results are available after only a relatively long time (4 h to 3 days). The identification of antibodies or viral antigens is robust, mainly because they rely on simpler technologies, but the low concentration of the targeted analyte in the sample decreases the sensitivity of the methods.^27^ Several approved diagnostics are based on colorimetric lateral flow assay (LFA), where the targeted analyte is detected using antibodies immobilized on a membrane. The advantage of LFA, compared to ELISA tests, is the possibility of using it at home without the need for personalized training, similar to the well-known pregnancy test, and the relatively low cost of the diagnostic. This is controversial for COVID-19 because typically used biological samples are extracted from nasal or oral swabs that must be collected by trained personnel to ensure the reproducibility of the test and guarantee a standardized collection procedure.^28,29^ In general, the sensitivity and specificity of PCR and LFA are high, but poorer performance is achieved when the viral load is too low to be detected (Figure 2), viz., when COVID-19 is still in its early stages. Even though the common testing procedures still require direct contact with the patient and trained staff for specimen collection, steps forward to ideal self-sampling and self-testing have recently been made. In the US and more recently in Europe, some home-tests have been authorized by the FDA under EUA. EmpowerDX and LabCorp are at-home COVID-19 RT-PCR tests containing a kit for the collection of the shallow, pain-free nasal sample that is then shipped back to the laboratory, and the results are available on the online portal between 24 and 48 h. In Germany and Spain, EmpowerDX PCT tests based on saliva or gargle samples are currently available. Ellume Limited is launching the first rapid COVID-19 at-home self-test on the US pharmaceutical market. The kit contains a nasal swab, and the diagnostic that analyzes the sample transmits the result automatically to the user’s smartphone via Bluetooth.

Anyway, as the demand for testing is constantly increasing, more burden on the laboratories prolongs to time to the test result. The lack of universal standardization increases this burden, as it requires each country to define its own policy. This influences the actual discovery rates of positive cases in the population, and threatens the path forward to gain control of the disease. For these reasons, healthcare systems worldwide require tests that are noninvasive, rapid, inexpensive, and easy-to-use tools for prescreening or ruling out infection at earlier stages, even before symptoms of COVID-19 manifest, before the well-accepted molecular confirmatory tests to decrease the virus spread and the mortality rates.

Nanotechnology has been used to develop biosensors for detecting SARS-CoV-2, as well as to improve RNA sequencing and make PCR technology affordable, easy to use, and portable.^30−33^ New strategies have been deeply revisited by other authors,^2,31,32,34^ and the detection accuracy of the methods available in the literature has been analyzed by meta-analysis.^33^Table 1 summarizes the pros and cons of the three more recurring techniques used in designing novel SARS-CoV-2 detection methods, exploiting the advantages of nanotechnology (i.e., magnetic, electrochemical, and optical methods), noting some examples. So far, the majority of these detection techniques can be used with samples from the respiratory tract, sputum and fecal specimens, with the exception of serological tests which require blood samples. Among all of them, nasopharyngeal and oropharyngeal swabs give the gold standard specimen for the diagnosis of SARS-CoV-2 due to the high viral load in the upper respiratory tract after onset of symptoms.^35^ Sputum (saliva), on the other hand, contains SARS-CoV-2 and therefore represents a valuable alternative for the diagnosis of COVID-19.^36^ The sensitivity and limit of detection (LOD) of COVID-19 diagnostics are determined by the infectious dose (= number of virus particles that are sufficient to infect 50% of a given population, the ID50) and the minimum viral load (= number of virus particles in an infected individual). Unfortunately, as of now, lack of knowledge on the infectious dose of SARS-CoV-2, as well as the variability of the viral load, make the comparison between the different diagnostic methods difficult. Current “best-in-class” diagnostic tests have detection limits of ∼100 copies/mL.^20^ However, due to the lack of standard protocols for sample collection and the possibility of personal errors, several studies reported low reproducibility and accuracy of tests.^37^

**Table 1:** Summary of Advantages/Limitation of Three Nanotechnology-Based Detection Approaches Commonly Used in Designing Novel COVID-19 Diagnostics

techniques	advantages	limitations	examples
Magnetic sensors^38^	Simple analyte isolation	Sample preparation	RNA extraction with magnetic beads^39^
	Improved signal/noise ratio	Time-consuming	Magnetic isolation and fluorescent detection^40^
Electrochemical sensors^41^	High sensitivity	Short self-life and limited stability over time	Magnetic isolation to improve electrochemical immunosensors^42^
	Rapid detection (between 20 to 45 min)^42,43^	Interferences to the signal	Fast SARS-CoV-2 detection using functionalized graphene electrodes^43^
	Possible miniaturization		Portable ultrasensitive electrochemical-base detection^44^
Optical sensors^45^	High sensitivity	High cost and development of POD challenging	Fluorescent-based nanoPCR using dual-functional magneto-plasmonic nanoparticles method^46^
	Rapid detection (between 10 and 20 min)^45^		Colorimetric and fluorescence signal LFA for semiquantitative and quantitative detection by smartphone-based device.^47^
			Label-free detection of SARS-CoV-2 using gold-nanoplasmonic sensor.^48^

## Airborne Transmission of SARS-CoV-2

**Figure 3:**
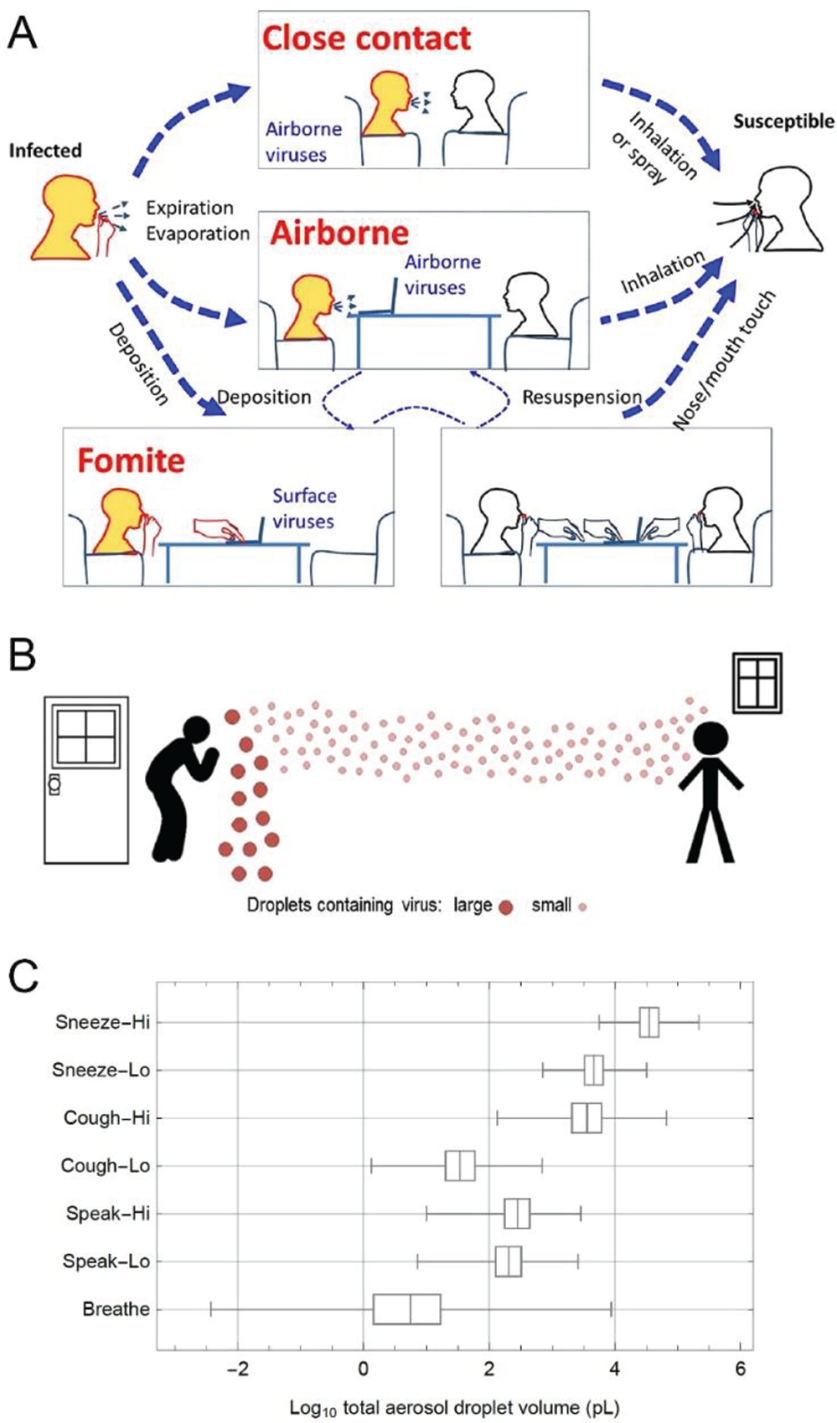
(A) Scheme of different possible transmission routes of SARS-CoV-2 through expiration (i.e., breathing, coughing, sneezing). Besides the close range and airborne transmission, virus-containing droplets can settle on surfaces (fomites, leading to self-inoculation). (Reproduced with permission from ref (78), Springer Nature). (B) Air diffusion of large and small virus-containing droplets. (Reproduced with permission from ref (57), Elsevier Ltd.) (C) Box-whisker chart of log10 of aerosol droplet volume (pL = picoliters). Box – median values; whisker – minimum and maximum values. The volume is considered as pL/20 min of breathing and speaking, and as pL per cough and sneeze (Reproduced with permission from ref (76)).

It is generally considered that viral respiratory infections spread by person-to-person transmission, and contact with contaminated surfaces is among the main routes to spread COVID-19 (Figure 3A).^49−51^ However, the high transmission rate of SARS-CoV-2 suggested that direct contact is not the only way of viral spreading, and virus-containing exhaled droplets have a fundamental role in the fast spread of infection.^52−55^ Some studies have confirmed the airborne transmission of COVID-19 through saliva droplets,^35,56^ whereas others have established dynamic flow models of airborne particles containing SARS-CoV-2 trying to elucidate the contexts in which COVID-19 airborne transmission mainly occurs.^57,58^ Two factors are considered in evaluating the airborne transmission: (i) the viral load in saliva and mucosae droplets, and (ii) the survival rate of the SARS-CoV-2 in the environment. It has been proven that the viral load can vary depending on the specimen being considered. SARS-CoV-2 is currently isolated from respiratory samples such as sputum and nasal and throat swabs/washes, with typical viral load ranging from 641 to 1.34 × 10^11^ copies/mL, with a median of 7.99 × 10^4^ copies/mL in throat samples, 10^5^ copies/mL in sputum, and 1.69 × 10^5^ copies/mL in nasal samples.^59,60^ Sneezing and coughing large drops of saliva and small drops from mucosae into the environment constitutes a high risk of infection. This risk is related to the viral load in the single drop (in turn relative to the droplet size) as well as to the number of droplets and their diffusion in the environment. It has been observed that more drops are released than while breathing normally, but the drops are the same size.^61,62^ The airborne transmission of COVID-19 can occur by inhalation of microscopic aerosol particles consisting of evaporated respiratory droplets, which are small enough to remain airborne for hours (<5 mm).^63^ Indeed, when infected individuals cough or sneeze, droplets containing SARS-CoV-2 are released. The larger droplets (>5–10 μm) fall on nearby surfaces, whereas the small ones (on the order of 1 μm) can remain airborne as aerosol and are breathed in by other people (environment-to-person transmission), as illustrated in Figure 3B.^61,64^ The airborne transmission route has been evaluated by means of theoretical models^64^ and studies of physic dynamics,^61,62,65^ while experimental evaluations are limited by the low viral load (<1 gene copies/m^3^).^50^ Other studies stated that a typical sneeze and cough could contain 40,000 and 3,000 droplets, respectively, leading to the spread of 10,000 to 2 × 10^8^ virosomes, depending on the viral load of the carrier.^66,67^ Doremalen et al. showed that the infectious titer (TCID50) in aerosols (<5 μm) containing SARS-CoV-2 reduced from 10^5.25^ per mL to 10^2.7^ TCID50 per liter of air after 3 h of experiment, which is too low to be detected with any sensor.^68^ As introduced previously, another important factor for airborne transmission is the survival rate of the virus in the environment. Dynamic modelings, supported by lab results, have indicated that the rapid spread of SARS-CoV-2 is favored by its long resistance in the air.^52,57,69^ Goh et al. used empirically based molecular tools to calculate the intrinsic disorder for SARS-CoV-2. The results confirmed its high resilience in saliva, and proved its ability to remain active for long periods outside the body, even in hostile environmental conditions.^70^ Arguably, this peculiarity is responsible for the high level of contagion, since the harder shell protects the virion from inactivation.^71^

To summarize, the high survival rate of SARS-CoV-2 and its airborne contamination can explain its high transmission rate, and yet this raises other questions. Considering that asymptomatic and presymptomatic individuals do not cough or sneeze to any appreciable extent, how are they contagious, and how do they generate aerosols? To answer these questions, we refer to the findings of Yan et al.^72^ who have shown that sneezing and coughing are not required for influenza virus aerosolization. Visualization by simple laser methods^73^ shows that the droplets produced while speaking are 20–500 μm in diameter and smaller while breathing—something that does not settle easily but diffuses through the air and is particularly dangerous in COVID-19 transmission.^74^ This conclusion has been supported by studies showing that wearing masks and respecting social distancing limit COVID-19 spread, in both asymptomatic and infected individuals.^75^ On the other hand, Schijven et al.^76^ have developed a method to estimate the airborne contamination with SARS-CoV-2 particles during speaking, coughing, and sneezing in an indoor environment. The total volume of exhaled droplets was higher during sneezing and coughing compared to speaking and breathing for 20 min (Figure 3C). Importantly, their study showed that the probability of contagion is strongly related to the virus concentration (1% probability of getting infected if the concentration is <10^5^ per mL). Netz at al.^77^ developed an equation describing the physical fate of droplets containing-SARS-CoV-2 produced while speaking, which depends on several parameters (size, relative humidity, temperature). Their results showed that when speaking, the virion concentration being exhaled increases, with an increase in droplets size ranging from 3 to 2 × 10^5^ virion per min for 1 to 40 μm droplet size, respectively. Standnytskyi et al. estimated, however, that at a saliva viral load of 7 × 10^6^ copies/mL, the probability that a 1 μm droplet nucleus (hydrated 3 μm droplet) contains a single virion is only 0.01%. However, if the titer is higher by 2–3 orders of magnitude, the number of exhaled virions in the emitted droplets can be expected to be ≫10^5^ per min of speaking.^77^ Although different tests have reported values that can span several orders of magnitude, it is clear from the above discussion that thousands of virions are emitted from infected people during normal breathing. Based on this statement, we raise the following question: “Can it be possible to develop a sensor to detect virions directly from exhaled breath without amplification and long sample treatment?”.

## COVID-19 Detection from Exhaled Breath

The analysis of exhaled breath could be a less invasive method of analysis for COVID-19 screening.^11,12,79,16^ Unfortunately, to date it has been extremely challenging to detect SARS-CoV-2 from exhaled breath. SARS-CoV-2 can be detected in air^80−83^ and objects that could affect the air around them (e.g., ventilation fans^84^ and on hospital floors^84^), mainly because the virus remains viable in the air for up to 3 h.^68,84^ Of special importance, parts of these studies^80^ show that COVID-19 patients exhaled millions of severe acute respiratory syndrome coronavirus RNA copies per hour. Experimental analyses show that exhaled breath had a higher positive rate (26.9%) than surface (5.4%) and air (3.8%) samples. Again, this emphasized the importance of aerosol transmission in virus spread. However, in order to detect the virus directly from exhaled breath, it was necessary to collect the sample for a long time with a specific method and technology called exhaled breath condensate (EBC). As demonstrated in recent papers, collecting and analyzing breath’s liquid phase (exhaled breath condensate or aerosol, EBC, and EBA, respectively), nonvolatile molecules such as RNA, DNA, microorganisms, and viruses can be directly detected (typically by means of successive PCR-based methods) and visualized.^85^ The use of EBC is related to the very low viral load in the breath. However, the viral load of SARS-CoV-2 in aerosol samples is several orders of magnitude below those in nasopharyngeal swabs, making the detection of the virus from the air in close contact with positive/acute patients more challenging.^86^ The use of EBC^87^ solves this challenge by preconcentrating the virus and its metabolic byproducts in exhaled breath, as well as large droplets or small aerosol particles from the epithelial lining fluid to the level of detectable concentrations. Importantly, even nonvolatile markers are released in the breath as large droplets or small aerosol particles from the epithelial lining fluid, and can be assessed in the exhaled breath.^88,78^ An EBC device can efficiently collect different particles in relation to two parameters: (1) the number of collected particles compared to the total amount of particles in the air; or (2) the fraction of virus that remains viable after collection. Apart from chilling tubes (called R-tubes), isolating particles from the breath can be achieved by specifically designed filters for aerosols, with an electrostatic concentrator, etc. Challenges associated with this approach is that the collected aerosol sample is usually ∼1 mL,^89^ and the results are affected by the breathing protocol (e.g., how deep the breath is, etc.). Since the viral load is very low, sample collection from 10 to 1500 mL/breath should be carried for a long time (30 min), or the patient should be asked to cough rather than simply breathing.^18^

Studies on exhaled breath showed that infection leads to a variation of the microbial flora in the lungs and, as a consequence, to a variation of exhaled metabolites. The variation of VOCs could be used to diagnose COVID-19 infection.^85,90^ In ref (60), for instance, the authors designed a method for direct detection of the virus, as well as related C-reactive protein and IgG and IgM markers, which, respectively, indicate the severity and immune response of the disease. While the detection of SARS-CoV-2 in saliva could be advantageous in terms of sample collection compared to nasopharyngeal sampling, the signals obtained are close to blank signal (sample/blank signal ratio 2.8–16). Grassin-Delyle et al.^9^ measured very specific VOCs in exhaled breath from mechanically ventilated adults with COVID-19 and compared that signature to ventilated patients with non-COVID acute respiratory distress syndrome. VOC-based breath signatures of COVID-19 could be distinguished from control cases with high accuracy. With this in mind, we think that the analysis of VOCs in breath has the potential to detect ketogenesis and other hematologic conditions related to SARS-CoV-2 infection, ensuring rapid detection and noninvasive sample collection. The rationale behind this approach relies on findings showing that viral agents and/or the body response (e.g., immune system) to the infectious/viral agent emit VOCs into the exhaled breath.^11,12^ The presence of VOCs in breath occurs in the early stages of the infection, thus serving for immediate detection of the COVID-19. The four most prominent VOCs in COVID-19 are methylpent-2-enal, 2,4-octadiene 1-chloroheptane, and nonanal, with typical concentrations of 10 to 250 ppb. Comprehensive reviews regarding the potential of VOCs as chemical biomarkers for disease diagnostics have been published.^12,11,91^

**Figure 4:**
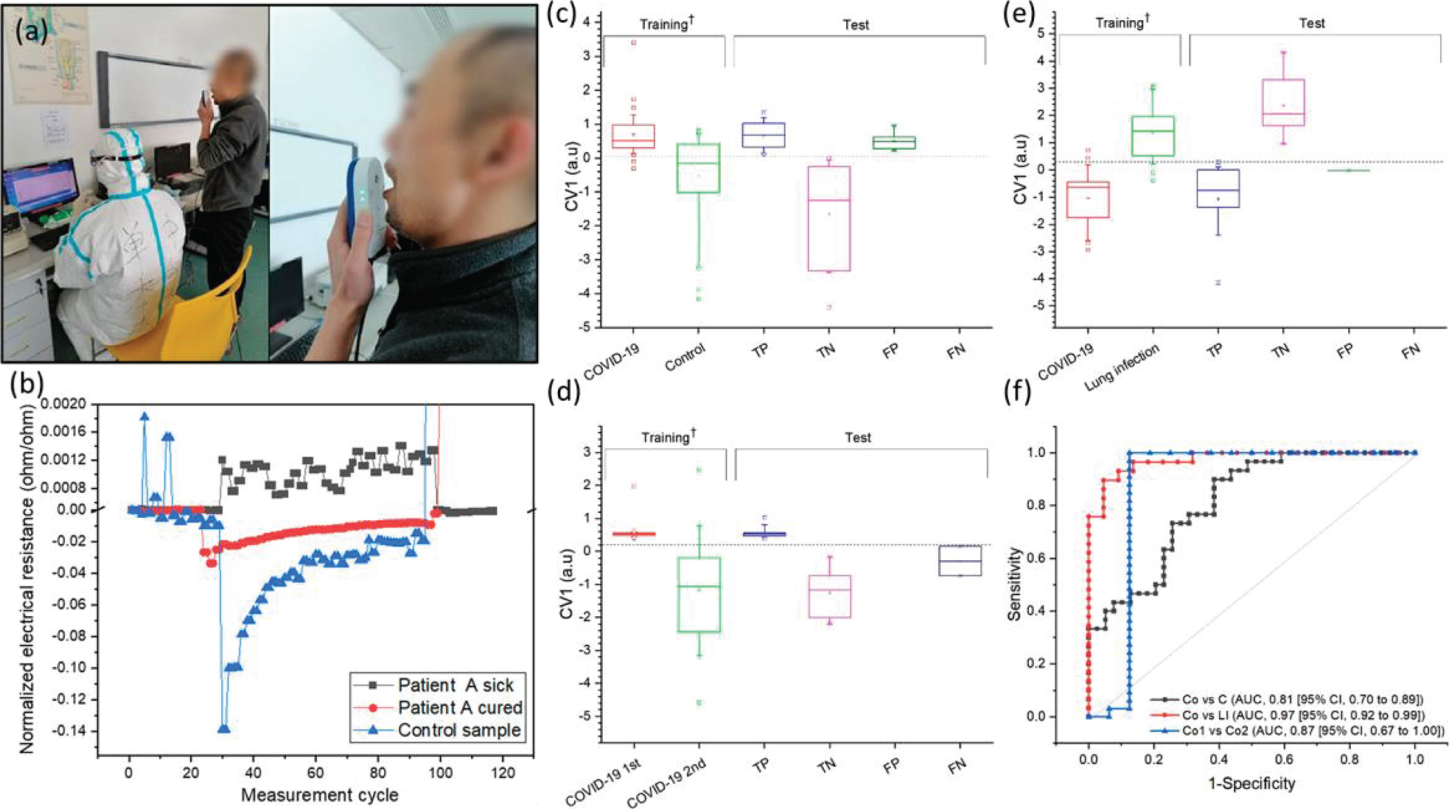
(a) Example of breath collection with the developed breathalyzer from a patient in Wuhan, China. (b) Representative response of a sensor to three different breath samples. The normalized response of the same in the breathalyzer to three different samples: patient A, COVID-19, first sample when infected; patient A, second sample after being determined as recovered; and healthy control. The *x*-axis represents the cycle measurement. (c–f) Diagnosis of COVID-19 patients based on breath sample response. Panels c, d, and e show data classification from sensor responses to breath samples as represented by the canonical variable of the discriminant analysis. Box plots of the first canonical score of the training set (70% of the samples) and test set (30% of the samples). The horizontal dashed line in the box plots represents the cutoff value of the model: true positive (TP), true negative (TN), false positive (FP), false negative (FN). (c) COVID-19 patients (*n* = 41) and healthy controls (*n* = 57). (d) COVID-19 patients (*n* = 41) and other lung infection/condition controls (*n* = 32). (e) COVID-19 patients at first (*n* = 41) and second sampling (*n* = 21). (f) ROC curves for the breath-sensor response in patients with COVID-19 (Co) infection compared with controls (C) (black); in COVID-19 infection compared with other lung infection/conditions (LI), (red); and in COVID-19 infection first sample (Co1) compared to COVID-19 infection second sample (Co2) (blue). ^†^*p* < 0.0001. (Reproduced with permission from ref (17) ACS Publications).

**Figure 5:**
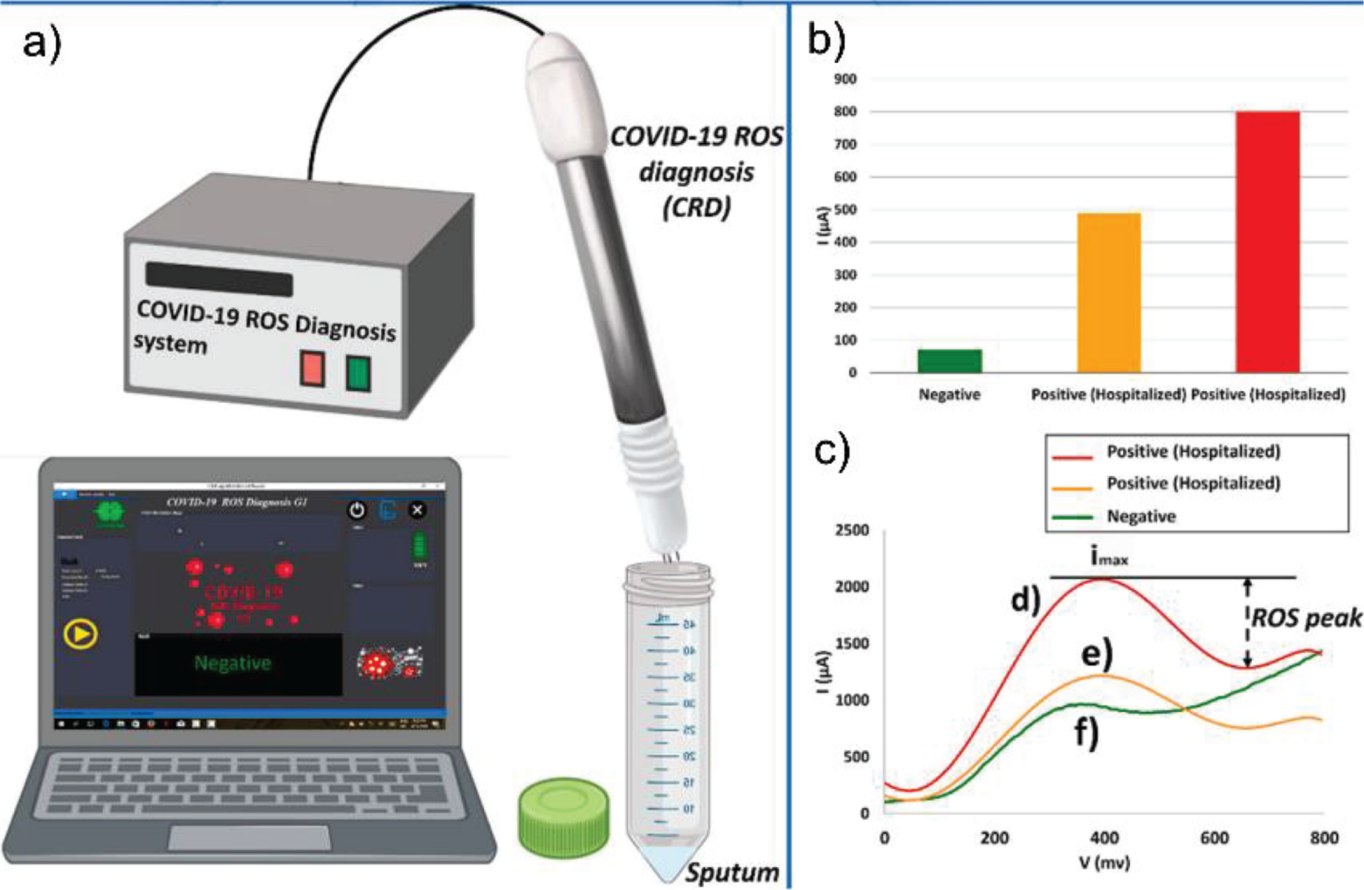
(a) COVID-19 ROS diagnosis (CRD) system consists of three needle electrodes coated with functionalized multiwall carbon nanotubes. (b) Selective electrochemical reactions of released ROS on MWCNTs produces cathodic ionic peaks. (c) ROS-related electrochemical cyclic voltammetry cathodic peaks from the fresh sputum of two different patients were involved in COVID-19 and hospitalized in comparison with a confirmed normal case. (Reproduced with permission from ref (92). Elsevier Ltd.).

In March 2020, Haick and co-workers^17^ concluded an exploratory clinical study in Wuhan, China (IRB: ChiCTR2000030556) that included sampling with a breath analyzer device based on an array of chemoresistive sensors made of molecularly modified gold nanoparticles in conjugation with machine-learning methods (Figure 4). The study cohort included 41 confirmed COVID-19 patients, 14 symptomatic negative COVID-19 patients, and 47 asymptomatic controls. Positive COVID-19 patients were sampled twice: (i) during active disease, and (ii) after cure of the disease. The Discriminant Factor Analysis (DFA) model achieved excellent training and blind discriminations between the different groups. For example, discrimination between (i) positive COVID-19 patients vs control resulted with 76% accuracy and 100% sensitivity; (ii) positive COVID-19 vs negative COVID-19 patients achieved 95% accuracy and 100% sensitivity; and (iii) positive COVID-19 patients before and after curing with 88% accuracy and 83% sensitivity (Figure 4).^17^ In another study, researchers monitored early traces of mitochondrial reactive oxygen species (ROS) elevated production as expressed in sputum samples.^92^ In this way, the introduction of sputum samples to an electrochemical sensor functionalized with multiwalled carbon nanotubes gave 97% true positive detection results within 30 s (Figure 5).

## Conclusions and Future Outlook

The recent COVID-19 pandemic has exposed the world to very serious challenges in fast diagnostics and monitoring of the outbreak. Selective sensing approaches that rely on specific and well-defined targets, such as in PCR, have been adopted toward fast diagnostics, but substantial pitfalls still exist. Indeed, such detection techniques are very disease-specific and their adaptation in the case of SARS-CoV-2 mutations requires significant effort and time. On the other hand, the use of a nonspecific sensing approach, mainly using breath samples, could go a long way toward healthful, responsible self-care.

We expect that breath-based detection methods, mainly online ones, will significantly reduce unnecessary exposure to contagious persons and support the fight against the COVID-19 pandemic. Moreover, it will reduce the number of excessive confirmatory tests and lower the burden on the hospitals, while allowing individuals a screening solution that can be used at home, PoC, and central facilities. The application of these approaches could incorporate secure data transmission components to enable ethical and privacy-ensured diagnosis and monitoring by physicians, national health systems, and worldwide health organizations. By creating a sample database, predictive models can be established for disease development among high-risk groups, regarding the hospitalization period and prognosis for positive patients. Breath-based approaches will enable adequate patient diagnosis, treatment, and follow-up, including continual screening of at-risk populations and real-time monitoring of epidemics. They will provide population-wide and location-based data for statistical analysis and data mining, and thereby facilitating the in-depth epidemiological study. They will also gather valuable information about future needs for infectious disease screening and monitoring among populations.

Using an advanced algorithm that merges deep analysis with powerful prediction capabilities from breath sensing platforms could help decision-makers and healthcare systems improve the way COVID-19 information is approached. This way, an integrated platform will enable continuous patient support, from predictive diagnosis to follow-up of COVID-19. It will reduce time, cost, and number of unneeded confirmatory tests, lowering the burden on hospitals. During hospitalization or home isolation, a breath analysis will serve as a monitoring tool for assessing the efficacy of treatment and disease regression. By creating a sample database, models can be established for predicting disease development among the high-risk groups, and hospitalization periods and prognosis for positive patients. The breath analysis platform will enable not only adequate patient diagnosis, treatment, and follow-up, but also continual screening of at-risk populations and real-time monitoring of epidemics. Although we think that the direct detection of SARS-CoV-2 virions from exhaled breath is not yet technologically possible, it is reasonable to develop new sensing devices that can effectively extract information from the exhaled breath to monitor patient status in real-time. In a world where everybody is wearing a face mask, the integration of a sensor on every single mask could radically revolutionize the monitoring of COVID-19 spread. A strong effort is needed to reach this goal, but the world community should be seeking this objective.
